# The analysis of competing hypotheses and expert witness testimony: Counteracting adversarial allegiance in witness credibility assessments?

**DOI:** 10.1371/journal.pone.0346891

**Published:** 2026-04-20

**Authors:** Jana Otzipka, Renate Volbert

**Affiliations:** 1 Freie Universität Berlin, Department of Education and Psychology, Berlin, Germany; 2 Psychologische Hochschule Berlin, Department of Forensic Psychology, Psychologische Hochschule Berlin, Berlin, Germany; Florida International University, UNITED STATES OF AMERICA

## Abstract

Cognitive biases, such as adversarial allegiance, can compromise expert witness evaluations and contribute to wrongful convictions. Therefore, the application of debiasing strategies is essential. The Analysis of Competing Hypotheses (ACH) has been proposed as a potential method to reduce such bias, although empirical support remains limited. The present study investigated the effectiveness of the ACH method in mitigating adversarial allegiance in a sample of mock expert witnesses for credibility assessments. In an online experiment, 159 participants with prior knowledge in credibility assessments reviewed a summary of a child sexual abuse case. Before reviewing the case material, participants were randomly assigned to one of three retaining party conditions: defense, accessory prosecution, or court. Next, half of the participants were instructed to apply the ACH method which includes the systematic comparison of alternative hypotheses within a matrix. Meanwhile the control group was instructed to follow the standard approach in credibility assessments in Germany, which includes the evaluation and falsification of alternative hypotheses, albeit in a less structured way than the ACH method. Outcomes were assessed using credibility ratings and an Evidence Score, the latter reflecting the extent to which participants weighed evidence in favor of their retaining party or evaluated information more evenly. No significant differences emerged in credibility ratings across conditions. However, adversarial allegiance was evident in the Evidence Score: defense-retained participants emphasized evidence undermining the statement’s credibility more than those retained by the accessory prosecution. At the same time, the application of the ACH method did not significantly influence credibility ratings or evidence selection. Overall, these findings suggest that the ACH method may have limited utility as a debiasing strategy in the context of credibility assessments and underscore the challenges of mitigating bias in forensic decision-making.

## Introduction

Credibility assessments play a critical role in criminal proceedings and can be instrumental in determining whether a defendant is to be convicted or acquitted [1]. As in other areas of forensic science, the assessments rely on the interpretation and evaluation of data by (psychological) expert witnesses [2]. The inherent room for interpretation introduces a degree of subjectivity, thereby elevating the potential for distorted judgments and biased evaluations [2]. Within the realm of (psychological) expert witness assessments, a range of cognitive biases can come into play [3], however, this study will specifically concentrate on adversarial allegiance, also recognized as allegiance bias. Adversarial allegiance can arise prominently, though not exclusively, within adversarial legal systems and holds the capacity to notably impact psychological expert witness evaluations [2,4].

When an expert witness is retained by one of the two opposing parties in adversarial legal systems – defense versus prosecution – various cognitive processes can contribute to the development of adversarial allegiance [5]. Firstly, being retained by either party in an adversarial process can foster a sense of team cohesion. Secondly, unconscious cognitive processes – reminiscent of tunnel vision [6] – may cause expert witnesses to emphasize evidence that aligns closely with the position of their retaining party, while disregarding contradictory evidence. Lastly, expert witnesses are often financially dependent on their retaining party, potentially reducing their inclination to challenge the positions of those who retained them [5]. Both field and experimental laboratory studies suggest that adversarial allegiance has a detrimental impact on the impartiality of expert witnesses’ assessments [e.g., 4, 7–10]. Chevalier et al. [7] demonstrated that the way expert witnesses report and interpret Static-99R scores – an actuarial risk assessment tool used to estimate the likelihood of sexual and violent reoffending among adult male sexual offenders – varies based on the party that usually retained them for evaluations. Furthermore, in a study by Murrie et al. [4], it was found that expert witnesses assigned higher or lower risk scores on the PCL-R – a clinician-rated measure of psychopathic traits, widely used in clinical and forensic settings and often considered in assessments of violent recidivism risk – and Static-99R based on whether they believed they had been retained by the prosecution or the defense. This effect was more evident in evaluations involving the PCL-R, which is considered to be a less standardized assessment tool compared to the Static-99R [4]. Edens et al. [8] as well as Lloyd et al. [9] reported similar findings for PCL-R assessments conducted by expert witnesses appointed by either the Crown (i.e., prosecution), the court, or the defense in Canada. McAuliff and Arter [11] specifically illustrated within the context of credibility assessments in cases of suspected child sexual abuse (CSA) that expert witnesses were more inclined to testify in favor of the defense or the prosecution when the available evidence and information supported their respective positions. Furthermore, the occurrence of adversarial allegiance was noted particularly in ambiguous situations lacking substantial evidence that would unequivocally lead to a certain conclusion (e.g., significant external suggestive influences [11]).

Although cognitive biases such as adversarial allegiance do not manifest in every criminal case, its presence can adversely affect expert witness testimonies. Therefore, it is crucial for expert witnesses to apply appropriate and effective debiasing strategies to counteract the adverse effects of cognitive biases [1,12]. Various debiasing strategies have been proposed in the literature, many of which may be applicable to the context of credibility assessments and adversarial allegiance. These include, for example, the “considering the opposite” strategy, which serves as a corrective strategy [13]. A similar strategy is considering alternative hypotheses [14], which also forms the basis of the Scenario Approach [15]. Additional strategies include transparent documentation, as well as the use of and, when feasible, outsourcing of standardized assessments such as risk assessment tools and psychological evaluations [12].

To date, two studies have examined the effects of adversarial allegiance on credibility assessments as well as potential debiasing strategies to counteract these effects [16,17]. In an initial experiment, Sauerland et al. [17] demonstrated that adversarial allegiance can be induced by providing participants acting as expert witnesses with letters from different retaining parties (defense vs. public prosecution) that emphasized the respective party’s position. In two subsequent experiments, the authors found that neither instructing participants to consider both information supporting and information opposing the reliability of the witness statement, nor employing the Scenario Approach [15], providing participants with three alternative scenarios, were effective in mitigating adversarial allegiance [17]. Arbiyah et al. [16] expanded on the findings of Sauerland et al. [17] by sensitizing participants to the possibility of alternative scenarios before they read the case file, in contrast to Sauerland et al. [17], who introduced the alternative scenarios after the case file had been presented. However, results from the first experiment indicated that participants did not evaluate the case differently when presented with an alternative scenario compared to when no alternative scenario was provided. A second experiment replicated this finding and further demonstrated that attempts to induce adversarial allegiance through the use of retention letters from opposing parties (defense vs. public prosecution) were unsuccessful. Only after combining data from both experiments and conducting an exploratory joint analysis did the authors find a statistically significant effect of considering alternative scenarios on the assessment of the suspect’s guilt. Nevertheless, the observed effect size was very small [16].

In Germany, the Federal Court of Justice (BGH) established minimum standards for credibility assessments in 1999 to ensure the quality and reliability of expert witness testimonies in this field. These minimum standards include the requirement to explore and dismiss alternative hypotheses before considering allegations credible [18,19]. The minimum standards therefore combine the application of alternative hypotheses as well as a falsifying approach. However, the judgment of the BGH does not provide explicit guidelines on the specific procedures that experts should employ when examining the alternative hypotheses. The findings of Sauerland et al. [17] and Arbiyah et al. [16] could indicate that the minimum standards may not sufficiently counteract cognitive biases such as allegiance bias.

At least in theory, a promising debiasing method that has some similarities with the minimum standards formulated by the BGH in 1999, is the Analysis of Competing Hypotheses (ACH) [20]. However, this method offers a more structured approach, which delineates the procedures for experts and enhances transparency in their evaluations. Therefore, it’s applications in credibility assessments could offer advantages.

Heuer [20] introduced the ACH-method for use by intelligence analysts handling complex and challenging assessments. In this context, hypotheses encompass any conceivable explanation or inference. The method involves evaluating the gathered evidence against these hypotheses. However, rather than sequentially analyzing each hypothesis independently, the ACH-method enables the hypotheses to compete simultaneously, while the analyst gauges their likelihood based on the available evidence. This approach also allows the analyst to assess the diagnostic value of each piece of evidence.

The ACH-method comprises eight steps [20].

**Fig 1:**
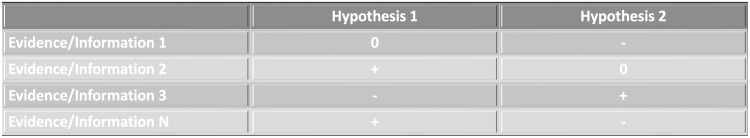
Example Matrix for Illustration Purposes.

1. First, all potential hypotheses should be identified. There is no set number of hypotheses to consider, but generally, the more complex the situation, the more alternative hypotheses should be considered. However, managing more than seven hypotheses becomes impractical.
2. In the second step, all the available evidence should be cataloged. Analysts should start with information that applies to all hypotheses and then consider the individual hypotheses, each leading to different questions being asked and evidence sought-out. Both the presence and absence of evidence should be documented.
3. Third, a matrix is created and analysts assess how well each piece of evidence aligns with each hypothesis across the rows of the matrix. This steps allows to evaluate the diagnosticity of each piece of evidence, pinpointing items most indicative of the hypotheses’ probabilities.
4. Fourth, the matrix is refined by removing non-diagnostic evidence and reconsidering the hypotheses. Evidence consistent with every hypothesis may lack diagnostic value entirely and should be removed from the matrix. Analysts should focus on highly diagnostic evidence for their final judgments.
5. During the fifth step, tentative conclusions on the hypotheses’ probabilities can be drawn, emphasizing falsification of the hypotheses rather than confirmation. Analysts should now focus on the columns of the matrix to gauge the hypotheses’ consistency with the available evidence. As Heuer [21 p. 4] states: “The most likely hypothesis is the one with the least evidence against it, not the one with the most evidence for it.” Fig 1 depicts an example matrix for illustration purposes.
6. The sixth step involves analyzing critical evidence as well as the previous tentative conclusions, considering potential errors or alternative interpretations.
7. In the seventh step, conclusions are reported and the relative likelihood of each possible interpretation is discussed.
8. Finally, in the eighth step, so-called “milestones” indicating unexpected developments that would suggest inaccuracies in the drawn conclusions are identified [20].

In essence, the ACH method appears to hold promise as a valuable tool for credibility assessments. The method integrates several debiasing strategies – including falsification, considering alternative hypotheses, and making reasoning explicit – and offers a transparent and structured way to organize diagnostic information compared to traditional, non-quantitative approaches [21–23]. At the same time, research on the ACH method’s effectiveness is scarce and its objectivity is debated, as ambiguous guidelines on the rating of evidence introduce subjectivity and potential bias [24]. Other concerns include the generality problem (i.e., how evidence is defined and individuated can shape the outcome) and the lack of rules for weighing the available evidence can lead to inconsistent judgments [23,25]. While empirical research on the ACH method remains limited [26], existing studies report mixed findings. Some studies suggest that the ACH method can mitigate confirmation bias in layperson samples [27] and enhance participants’ ability to accurately evaluate the diagnosticity of cues [28]. Moreover, the ACH method has been shown to support the correct identification of available cues [25]. In contrast, several studies have found no significant debiasing effects of the ACH method when addressing confirmation bias or serial position effects, particularly among experienced participants [27,29]. Yet other studies showed no improvements in the accuracy of evaluations [28,30], the search for and evaluation of the available evidence [31,32], the recognition of diagnostic evidence [32] or the coherence and consistency of judgments [33] when compared with control groups. Additionally, some studies indicate that participants may double-count cues when applying the ACH method [30] and display pseudo-diagnosticity [33]. Finally, confirmation bias has been shown to influence the ACH method’s scoring process [29]. Beyond these mixed and occasionally contradictory findings, many of the existing studies relied on relatively small sample sizes, limiting the robustness and generalizability of their conclusions. Furthermore, no research to date has examined the applicability of the ACH method to expert witness testimonies in general or to credibility assessments of eyewitness statements in particular.

The present study seeks to address the described research gap by answering the following research question: Can the implementation of the ACH method enhance evidence evaluation and decision-making by (mock) expert witnesses in credibility assessments while counteracting allegiance bias? The ACH method was evaluated within the German legal context, which presents a unique setting. As outlined in previous sections, the 1999 BGH ruling established that expert witnesses conducting credibility assessments are required to consider alternative hypotheses and apply a falsification-based approach. Accordingly, the present study aims to examine whether the structured framework of the ACH method can offer advantages over the traditional, non-quantitative approach that has been in use in Germany for more than 25 years.

### Hypotheses

To test whether the ACH method is effective in counteracting adversarial allegiance in (mock) credibility assessments the following hypotheses were formulated and preregistered on OSF (registration DOI: https://doi.org/10.17605/OSF.IO/BDN7G):

1. First, it was expected that participants will display allegiance bias and rate the credibility of the CSA allegations based on their retaining party. Only participants retained by the court will not display allegiance bias.
2. Second, it was predicted that participants who are instructed to use the ACH method will evaluate the statement’s credibility in a more balanced manner compared to the control group. Moreover, the application of the ACH method will have the strongest effect on participants retained by adversarial parties as opposed to the court (interaction effect).
3. Third, it was expected that participants will focus on the evidence that was emphasized by and is in-line with the position of their retaining party. Only participants retained by the court will focus on both kinds of information and evidence.
4. Fourth, the effect described under hypothesis 3 should be reduced when participants apply the ACH method, causing participants to consider both types of information irrespective of their referral source. Moreover, the ACH method will have the strongest effect on participants retained by adversarial parties as opposed to the court (interaction effect).

Additionally, the perceived difficulty of the case evaluation was explored in an exploratory manner. Due to the absence of prior research, it was not possible to formulate specific hypotheses regarding the impact of retaining party and the two evaluation methods on perceived difficulty.

## Methods

### Participants

Psychologists and psychology students with prior knowledge in legal psychology, specifically in credibility assessments, were recruited to ensure an accurate implementation of the case assessment and the potential applicability of the results to the intended target audience of the ACH method. Participants were recruited through personal networks, pertinent social media platforms, and university-specific research participation channels.

Participants were compensated for their participation in the study with a digital Amazon voucher, the value of which was based on their assigned group (€5.00 for the control group and €7.00 for the ACH group). To avoid potential confounding effects, participants were not informed in advance of the specific amount associated with their condition. Instead, they received this information, along with instructions on how to claim their compensation, only after completing their participation. Alternatively, participating psychology students from the Psychologische Hochschule Berlin (PHB) had the option to receive one research participation point upon request.

#### Sample size determination.

An a priori power analysis by means of G*Power [34] established that a minimum sample size of at least 206 participants was required (α = .05, power = .90, f = 0.25). The detailed power analysis is summarized in the published OSF preregistration (registration DOI: https://doi.org/10.17605/OSF.IO/BDN7G).

The selection of a medium effect size was based on two considerations: First, an effective debiasing strategy is expected to produce meaningful, and ideally large, effect sizes. However, due to the limited amount of prior research, it was not possible to derive a more precise estimate of the anticipated effect size. Second, practical considerations informed the choice of a medium effect size. The pool of potential participants in Germany with relevant background knowledge in credibility assessment is relatively small. Thus, the chosen effect size reflects a compromise between ensuring adequate statistical power and maintaining a feasible and realistic sample size.

Shortly after data collection began, it became evident that reaching the preregistered sample size of 206 participants would not be feasible. Although a sufficient number of individuals expressed a willingness to participate, an unexpectedly large proportion lacked the necessary background knowledge, despite theoretically possessing it – for instance, based on their previously obtained degrees in legal psychology. Consequently, a secondary power analysis was conducted during data collection, adjusting the required statistical power to .80 while keeping all other parameters unchanged. This analysis determined that a total sample of 158 participants would be sufficient.

#### Exclusion criteria and data screening.

Only participants with background knowledge in credibility assessments were eligible to take part in this study. Therefore, participants who reported having no prior knowledge in the field of legal psychology (*n* = 10) were excluded from the study at the outset. To further confirm participants’ requisite background knowledge, two multiple-choice questions assessing familiarity with standard procedures in credibility assessments were administered. The first question asked which of three listed alternative hypotheses is not routinely examined in credibility assessments. The response options were: the false memory hypothesis, the intentional fabrication hypothesis, and the repression hypothesis (correct answer). The second question asked how the potential presence of suggestive influences is examined and offered five response options: the motive analysis, the reconstruction of the history of the formation and evolution of the statement (correct answer), the statement’s quality as well as a the criteria-based content analysis, the quality-competence comparison, and the analysis of fabricated fantasy stories. Participants who answered one or both of these questions incorrectly had their participation in the study terminated immediately (*n* = 78). Moreover, to ensure robust data quality three multiple-choice questions related to the provided case file were included as attention checks. Participants whose responses to one or more of these questions were incorrect had their participation promptly concluded (*n* = 18). One of these attention-check questions assessed whether participants accurately identified their retaining party, thus serving as a manipulation check question. Only complete datasets were included for analysis purposes.

In total, complete datasets from 164 participants were collected between September 15, 2023 and the December 1, 2024. After removing five participants who reported not following the study instructions, the final sample comprised 159 participants.

#### Sample characteristics.

Participants ranged in age from 21 to 65 years (*M* = 33.5, *SD* = 9.1), with a median age of 31 years. The sample was predominantly female (85.5%), while 13.8% identified as male, and 0.6% reported a different gender identity. The highest level of education attained varied, with the majority holding a Master’s degree (59.7%), followed by a Bachelor’s degree (15.1%), a PhD (9.4%), and a high school diploma (3.8%; these participants were currently Bachelor’s students). Furthermore, 6.3% (*n* = 10) were certified specialist psychologists in legal psychology, and 5.7% (*n* = 9) were certified psychological psychotherapists. At the time of data collection, most participants (54.7%) were currently enrolled in academic programs, with their semester of study ranging from the 1st to the 10th semester (*M* = 3.7, *Mdn* = 3, *SD* = 2.2).

### Design

The study employed a 2 × 3 between-subjects design, with instruction type (ACH group vs. control group) and retaining party (accessory prosecution vs. defense vs. court) as independent variables. Participants were randomly assigned to one of the six resulting groups.

### Procedure

An online study was created using the online survey tool SoSci Survey (https://www.soscisurvey.de) to address the research question. Approval for the study was obtained from the Ethical Committee of the PHB (no. EK2022/09). Participants were given detailed information about the study and provided written informed consent at the beginning of the survey.

Next, participants were provided with a summary of a phone call and a letter inviting them, as expert witnesses for credibility assessments, to offer an expert opinion on a suspected case of CSA. The retaining party was varied, serving as the first independent variable in the study design. Each phone call and letter emphasized the position and expectations of the respective retaining party. Given Germany’s inquisitorial legal system, where the public prosecutor’s office is perceived as more neutral compared to adversarial legal systems, the study specified the following retaining parties: the defense, the accessory prosecution (in German termed “Nebenklagevertretung”), and the court. The accessory prosecution permits victims of serious crimes, including sexual offenses, to actively participate in the criminal proceedings against the defendant alongside the public prosecutor. Usually, victims are represented by legal counsel who assumes the role of accessory prosecutor. This representative may support the prosecution or contest decisions if it appears that the public prosecutor is not adequately representing the victim’s interests. The court was included as a control group, representing a neutral retaining party. The contrasting positions of the defense and the accessory prosecution aimed to provoke the emergence of adversarial allegiance, allowing for an examination of the potential beneficial effects of the ACH method as a debiasing method in this context.

Following the participants’ review of the letter from their respective retaining party, they all received an identical case file summarizing relevant information about a criminal investigation concerning a suspected case of CSA. The case presented was intentionally ambiguous, including information that both supported the credibility of the allegations as well as information that made it challenging to dismiss alternative hypotheses. Accordingly, there was no objectively correct assessment outcome. Instead, the ambiguity of the case created an opportunity for cognitive biases, specifically adversarial allegiance, to influence the weighing and selection of information underlying participants’ evaluations.

Subsequently, participants were divided into a control group and an ACH group within each retaining party condition. This subdivision in terms of instruction served as the second independent variable.

#### Control group.

The control group was instructed to proceed with their evaluation of the case in the standard manner, while being reminded of the minimum standards for credibility assessments established by the German Federal Court of Justice in 1999 [18], which requires the consideration of alternative hypotheses. At the conclusion of their participation, participants in the control group were asked whether they had evaluated alternative hypotheses during their case assessment.

#### ACH group.

Conversely, the ACH group received instructions on how to utilize the ACH-method. Aligned with the minimum standards for credibility assessments [18], the ACH matrix specified the hypotheses to be evaluated as follows:

1. Hypothesis 1: The alleged victim’s account is based on genuine experiences, the reported CSA occurred.
2. Hypothesis 2: The alleged victim is deliberately falsely accusing the defendant, the reported CSA did not occur.
3. Hypothesis 3: The alleged victim’s statement is influenced by external suggestive factors, the reported CSA did not occur.

Next, participants were asked to extract all information from the case file summary that they considered relevant to assessing the three competing hypotheses and to enter these data into the ACH matrix (step 2 – ACH method). They were further reminded to consider the various aspects of credibility assessment – specifically, the quality, consistency, formation, and evolution of the statement – when identifying pertinent information within the case file summary. To proceed to the following section of the survey, participants were required to identify a minimum of three and a maximum of 20 distinct pieces of information. Each selected item of information was then assessed in relation to the three hypotheses. Participants indicated a “+” when the evidence was consistent with a given hypothesis, a “−” when it was inconsistent with the hypothesis, and a “0” when it was neutral or unrelated (step 3 – ACH method). Subsequently, participants were instructed to eliminate non-diagnostic evidence from the matrix – that is, information evaluated identically across all hypotheses (step 4 – ACH method). A brief rationale was provided to clarify why such evidence is considered non-diagnostic. Once removed, these items were no longer visible during the subsequent analytical steps. Next, participants evaluated the hypotheses in terms of their overall consistency or inconsistency with the remaining information (step 5 – ACH method). At this point, they were instructed to apply the principle of falsification by favoring the hypothesis that accumulated fewer inconsistent (“−”) ratings rather than the one with the most consistent (“+”) ratings. The accompanying explanation emphasized that the frequency of inconsistencies provides a more informative and reliable indicator of validity than the number of consistent findings. Finally, in formulating their overall conclusions, participants were advised to integrate both the outcomes of their matrix analysis and their own judgment regarding which pieces of evidence they considered most significant.

#### Final measurements.

Lastly, participants across all groups were asked to indicate how likely they considered the allegations of CSA to be credible and based on genuine experiences. They were also requested to provide their conclusion(s) in their own words (a single sentence sufficed). Additionally, participants were asked to identify the information they deemed most significant and compelling in their evaluation process. The ACH group selected this information from their matrix. The control group – who had not previously provided a list of analyzed information – were now also required to report at least three and no more than 20 pieces of information they considered most influential. Finally, participants reported their perceived difficulty in evaluating the case.

The average completion time for the survey was 31.79 minutes, with individual completion times ranging from 299 to 3,627 seconds.

### Dependent variables

Participants indicated the perceived probability of the credibility of the allegations using a slider bar, with a scale ranging from 0 (very unlikely) to 100 (very likely).

Additionally, an Evidence Score was calculated for each participant to evaluate the extent to which their selection of evidence was influenced by the retaining party. After data collection, each item of evidence or relevant information that participants selected as most compelling in their own evaluation was coded (for more information regarding the coding see the section on interrater reliability, below). The assigned value reflected the piece of information’s alignment: −1 for defense-oriented, +1 for accessory prosecution-oriented, and 0 for neutral. Based on the coded values, an individual Evidence Score was computed by summing the values of the pieces of evidence each participant identified as the most compelling. To account for variations in the number of selected most compelling pieces of evidence, the sum score was divided by the total number of such selections made by each participant. Consequently, the resulting Evidence Score ranges from −1 to +1. Participants who focused predominantly on evidence and information consistent with the defense attorney’s position received an Evidence Score < 0. Conversely, participants who focused primarily on information and evidence in line with the position of the accessory prosecution received an Evidence Score > 0. Lastly, participants who considered both types of evidence received an Evidence Score close to 0.

Finally, participants’ reported perceived difficulty in evaluating the case was measured on a scale from 0 (not difficult) to 100 (very difficult).

### Interrater reliability

To calculate the previously described Evidence Score based on participants’ selections of the most compelling pieces of evidence, all evidence and information identified and listed by participants were coded according to their alignment with the respective retaining parties. For this purpose, a coding manual was developed. Each item could be classified as defense-oriented, accessory prosecution-oriented, or neutral. The manual also provided three examples each for defense-oriented and accessory prosecution-oriented evidence to guide consistent coding. Additionally, coders could indicate whether a piece of information was mixed (i.e., contained elements aligning with more than one retaining party), incorrect, or unclear. In the control group, coding decisions were based solely on the wording of the selected evidence. In contrast, for the ACH group, coding could also draw on participants’ ACH matrix evaluations. The coding manual included specific instructions for how to interpret various combinations of consistency ratings in these ACH matrices.

In the control group, participants listed *M* = 5.53 (*Mdn* = 5) items as the most compelling pieces of information from the case file. In the ACH group, participants selected *M* = 3.95 (*Mdn* = 4) items as most influential from the *M* = 8.53 pieces of information (*Mdn* = 7.50) they had previously identified as relevant.

To ensure coding reliability, all participants’ responses were independently coded by two raters: the first author and an additional coder who was blind to the study’s design and hypotheses. Interrater reliability, as measured by Cohen’s Kappa, was κ = .775.

## Results

### Credibility ratings (hypotheses 1 and 2)

To provide an overview, Table 1 lists the descriptive statistics for the credibility ratings according to each sub-group within the study design.

**Table 1:** Descriptive Statistics for Credibility Ratings Measured on a Scale From 0 to 100.

Evaluation method	Retaining party	M	SD
Control group (*n* = 73)	Accessory Prosecution (*n* = 23)	27.61	17.46
	Court (*n* = 26)	24.23	12.96
	Defense (*n* = 24)	21.54	15.09
ACH group (*n* = 86)	Accessory Prosecution (*n* = 26)	26.81	25.08
	Court (*n* = 29)	31.55	22.49
	Defense (*n* = 31)	19.00	12.89

As preregistered, an independent factorial ANOVA was conducted to examine the effects of evaluation method and retaining party on credibility ratings (Hypotheses 1 and 2). A residual analysis was performed to assess the assumptions of the factorial ANOVA. Outliers were identified through an examination of boxplots, revealing a total of seven outliers. However, none were classified as extreme outliers. Notably, all outliers were found within the ACH group. To ensure a comprehensive analysis, these outliers were retained in the dataset. The normality of residuals was evaluated using Shapiro-Wilk’s normality test for every cell of the design. The residuals deviated significantly from normality (*p* < .050) in all but one subgroup: participants retained by the court in the control group (*p* = .380). However, given that the factorial ANOVA is generally robust to violations of normality, particularly when all groups exhibit similar distributions – in this case, a right-skewed distribution – and the sample size is sufficiently large, this deviation was not considered problematic. Additionally, Levene’s test for equality of error variances, applied to the median due to the skewed distribution, indicated homogeneity of variances (*p* = .108).

Contrary to expectations, the interaction effect between evaluation method and retaining party on credibility ratings was not statistically significant, *F*(2, 153) = 1.12, *p* = .328, partial η2 = .014. Consequently, main effects were analyzed. Neither the main effect of retaining party, *F*(2, 153) = 2.86, *p* = .060, partial η2 = .036, nor the main effect of evaluation method on credibility ratings, *F*(1, 153) = .21, *p* = .649, partial η2 = .001, reached statistical significance.

**Fig 2:**
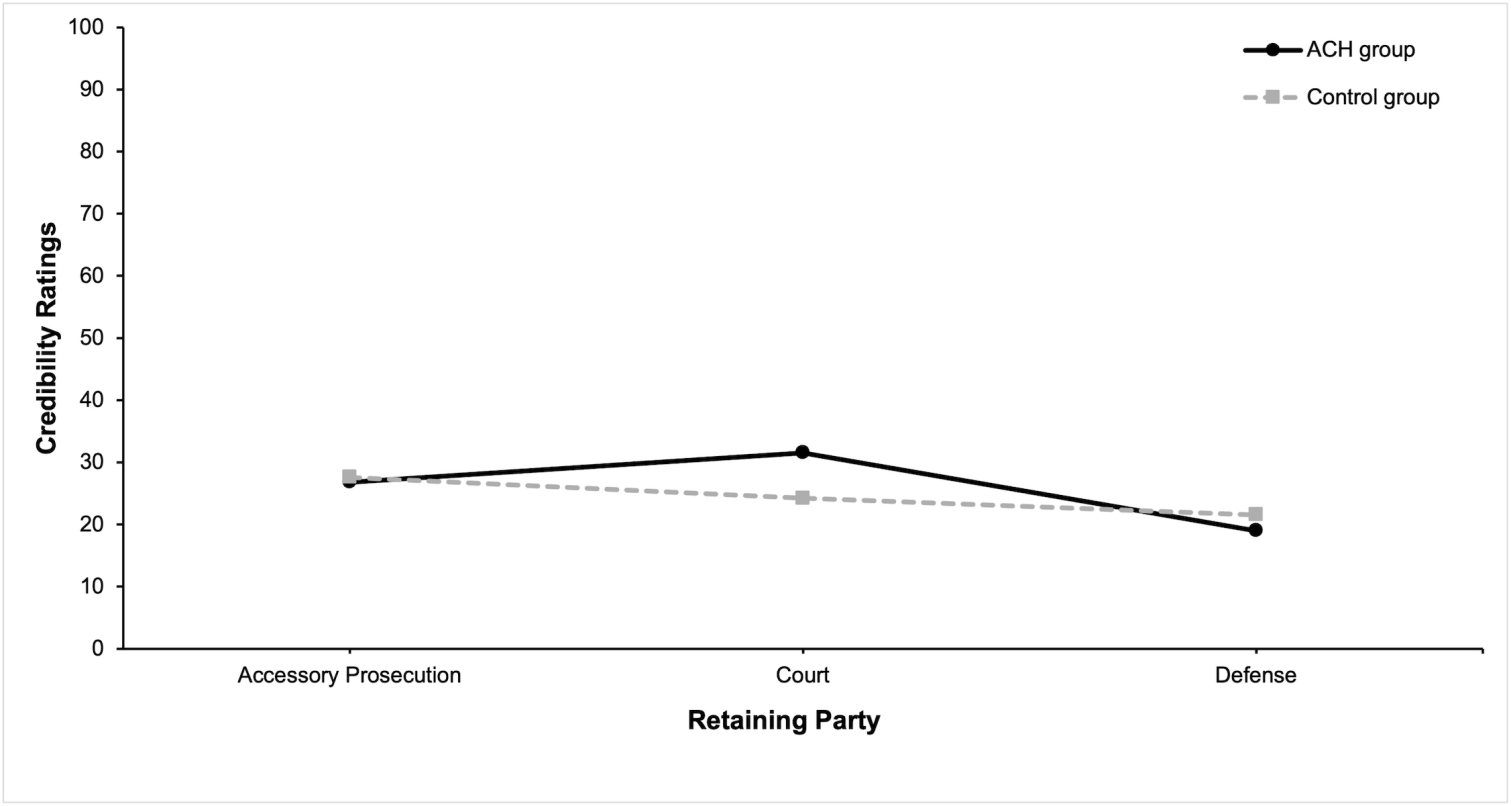
Credibility Ratings Across Retaining Parties and Evaluation Methods.

To conclude, neither Hypothesis 1 nor Hypothesis 2 was supported. Fig 2 depicts the distribution of credibility ratings based on the different retaining parties and evaluation methods.

### Evidence Score (hypotheses 3 and 4)

Table 2 provides an overview of the descriptive statistics for Evidence Score across the different subgroups within the study design.

**Table 2:** Descriptive Statistics for Evidence Score Measured on a Scale From −1 to +1.

Evaluation method	Retaining party	M	SD
Control group (*n* = 73)	Accessory Prosecution (*n* = 23)	−.66	.41
	Court (*n* = 26)	−.72	.36
	Defense (*n* = 24)	−.80	.30
ACH group (*n* = 86)	Accessory Prosecution (*n* = 26)	−.71	.52
	Court (*n* = 29)	−.78	.45
	Defense (*n* = 31)	−.94	.17

As specified in the preregistration, a second independent factorial ANOVA was carried out to investigate the impact of evaluation method and retaining party on Evidence Score (Hypotheses 3 and 4). To verify the assumptions of the factorial ANOVA, a residual analysis was conducted. Boxplots were used to detect outliers, revealing a total of eleven outliers, seven of which were classified as extreme. Notably, all outliers were again found within the ACH group. Further investigation, particularly of the extreme outliers, ruled out measurement or data entry errors. It was observed that five of the seven extreme outliers were participants retained by the defense in the ACH group. These five participants were the only ones in this subgroup who did not have an Evidence Score of −1.00, instead exhibiting scores between −0.25 and −0.80. The low variance within the ACH-defense group led to these values being flagged as extreme outliers. However, removing these data points would have led to the loss of valuable information regarding the true variation in the Evidence Scores. Therefore, all outliers were retained in the dataset. Next, residual normality was assessed using Shapiro-Wilk’s normality test for every cell of the design, revealing significant deviations from normality (*p* < .001) across all subgroups. An analysis of histograms indicated that all groups demonstrated a right-skewed distribution. Nonetheless, for the reasons already outlined in the previous section, the factorial ANOVA was considered robust to this violation of normality. Lastly, the Levene’s test for equality of error variances, applied to the median due to the skewed distribution, indicated homogeneity of variances (*p* = .110).

Contrary to expectations, the interaction effect between evaluation method and retaining party on the Evidence Score was not statistically significant, *F*(2, 153) = .03, *p* = .804, partial η2 = .003. Consequently, main effects were analyzed. The main effect of retaining party was statistically significant, *F*(2, 153) = 3.12, *p* = .047, partial η2 = .039, while the main effect of evaluation method on Evidence Scores was not, *F*(1, 153) = 1.79, *p* = .183, partial η2 = .012.

Post hoc testing with Bonferroni correction revealed that the Evidence Scores were significantly lower for participants retained by the defense compared to those retained by the accessory prosecution (*p* = .047, mean difference = −.19, 95% CI [−.37, −.00]). However, no significant differences were observed between participants retained by the defense and those retained by the court (*p* = .301, mean difference = −.12, 95% CI [−.30, .06]) or between participants retained by the accessory prosecution and those retained by the court (*p* = 1.000, mean difference = −.06, 95% CI [−.25, .12]).

**Fig 3:**
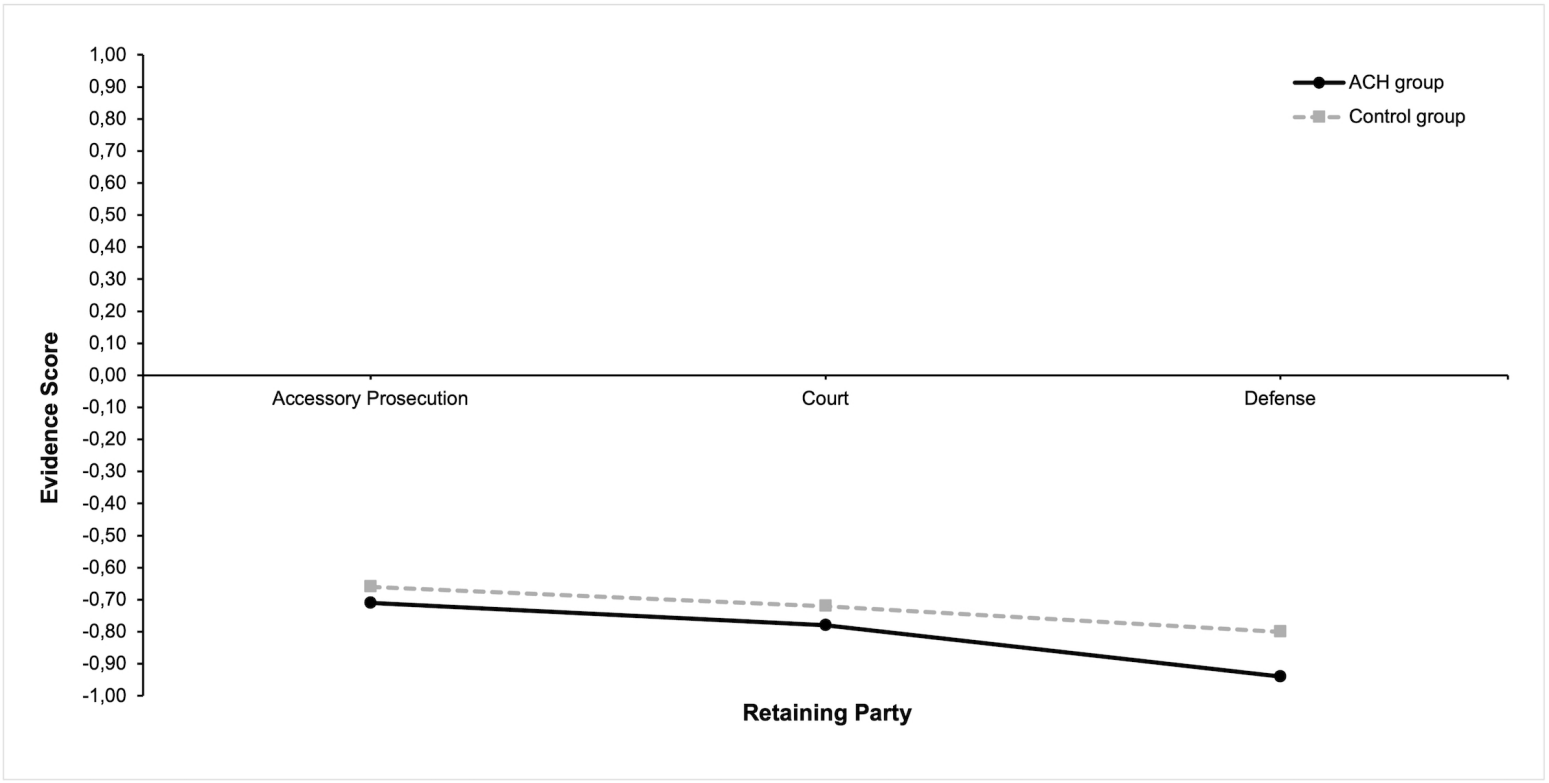
Evidence Scores Across Retaining Parties and Evaluation Methods.

In conclusion, Hypothesis 3 was partially supported, whereas Hypothesis 4 was not confirmed. Fig 3 illustrates the distribution of Evidence Scores across the different retaining parties and evaluation methods.

### Exploratory analysis

Given the lack of prior research, the perceived difficulty of the case evaluation was examined exploratorily, focusing on the potential influence of the different retaining parties as well as evaluation methods. Table 3 provides an overview of the descriptive statistics for the perceived difficulty in case evaluation across the different subgroups within the study design.

**Table 3:** Descriptive Statistics for the Perceived Difficulty in Case Evaluation Measured on a Scale From 0 to 100.

Evaluation method	Retaining party	M	SD
Control group (*n* = 73)	Accessory Prosecution (*n* = 23)	41.35	27.52
	Court (*n* = 26)	45.92	24.44
	Defense (*n* = 24)	34.00	28.86
ACH group (*n* = 86)	Accessory Prosecution (*n* = 26)	42.04	29.23
	Court (*n* = 29)	50.10	27.38
	Defense (*n* = 31)	35.48	28.19

A third independent factorial ANOVA was carried out to investigate the impact of evaluation method and retaining party on perceived difficulty in the case evaluation. Again, a residual analysis was conducted to assess the assumptions of the factorial ANOVA. Boxplots revealed no outliers while Shapiro-Wilk’s normality tests for every cell of the design revealed significant deviations from normality (*p* < .050) for all but two subgroups: participants retained by the court, both in the control group (*p* = .141) as well as the ACH group (*p* = .137) were normally distributed. Histograms showed that all groups not conforming to normality demonstrated a right-skewed distribution. As previously reasoned, the factorial ANOVA was considered robust to this violation of normality. Finally, the Levene’s test for equality of error variances, applied to the median due to the partially skewed distribution, indicated homogeneity of variances (*p* = .942).

The interaction effect between evaluation method and retaining party on the perceived difficulty was not statistically significant, *F*(2, 153) = .06, *p* = .944, partial η2 = .001. Subsequently, main effects were analyzed. The main effect of retaining party on the perceived difficulty was statistically significant, *F*(2, 153) = 3.14, *p* = .046, partial η2 = .039, however, the main effect of evaluation method was not, *F*(1, 153) = .23, *p* = .632, partial η2 = .002.

Post hoc testing with Bonferroni correction revealed that the perceived difficulty in the case assessment was significantly higher for participants retained by the court compared to those retained by the defense (*p* = .040, mean difference = 13.27, 95% CI [.45, 26.10]). However, no significant differences were observed between participants retained by the court and those retained by the accessory prosecution (*p* = .741, mean difference = 6.32, 95% CI [−6.85, 19.49]) or between participants retained by the defense and those retained by the accessory prosecution (*p* = .614, mean difference = −6.95, 95% CI [−20.16, 6.26]).

**Fig 4:**
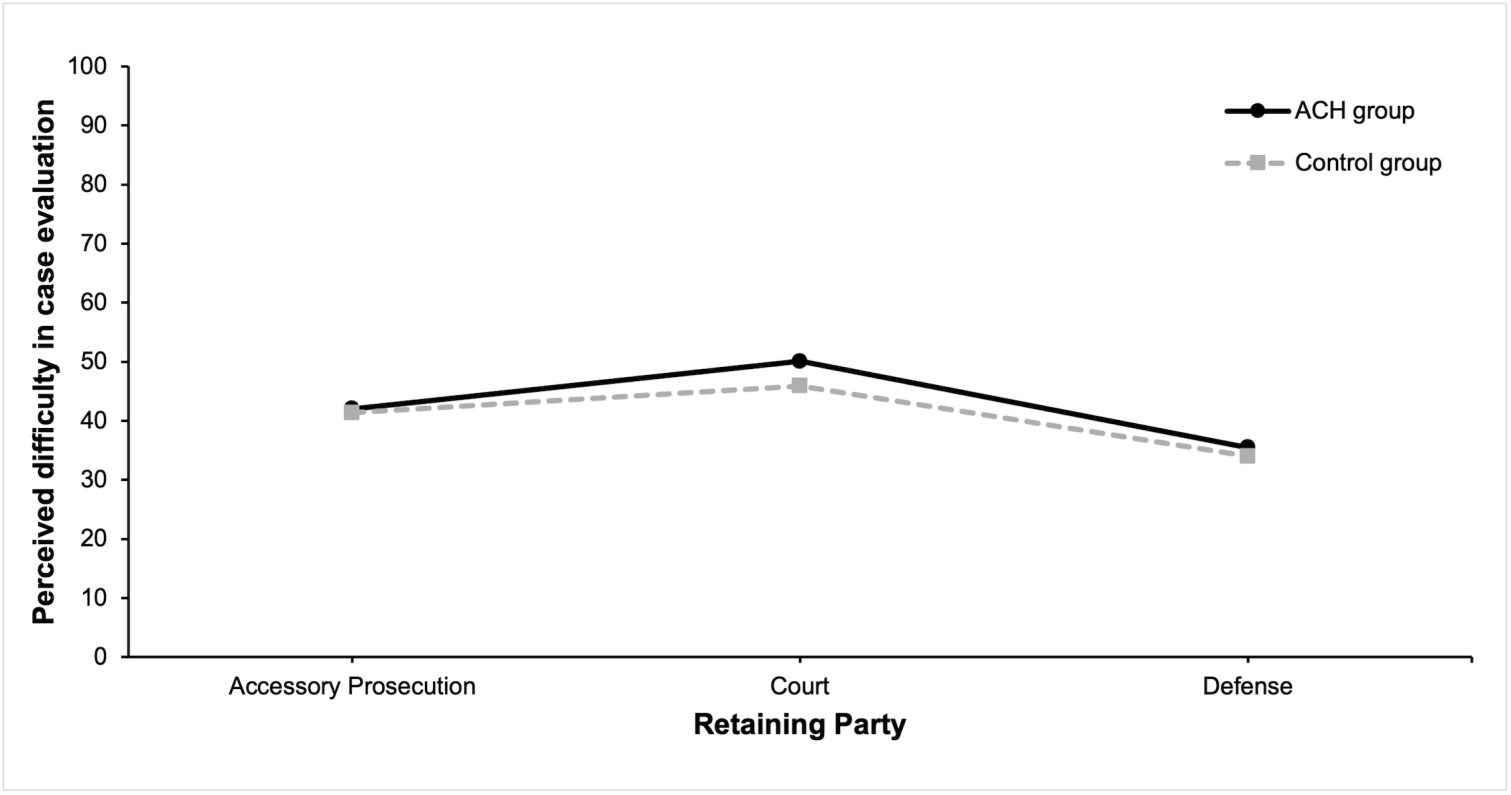
Perceived Difficulty in Case Evaluation Across Retaining Parties and Evaluation Methods.

Fig 4 illustrates the distribution of perceived difficulty in case evaluation across the different retaining parties and evaluation methods.

## Discussion

This study represents the first attempt to evaluate the applicability of the ACH method in the context of credibility assessments by (mock) expert witnesses in general, and as a debiasing tool against adversarial allegiance in particular.

With respect to the credibility ratings, no statistically significant evidence was found to support the emergence of adversarial allegiance. However, descriptively, the ratings within the control group followed the anticipated pattern. On the other hand, adversarial allegiance did emerge for the Evidence Score. Participants retained by the defense focused significantly more on evidence challenging the statement’s credibility compared to participants retained by the accessory prosecution. Nonetheless, the average Evidence Score was consistently negative across all three retention groups, ranging from *M* = −0.66 to *M* = −0.94, indicating that participants in all groups focused more on evidence that was critical of the statement’s credibility. In the context of credibility assessments in Germany, expert witnesses are required to rule out all possible alternative explanations, such as false testimony or false memories, before deeming a statement credible [18,19]. Thus, a critical evaluative approach is to be expected. Nevertheless, adversarial allegiance did influence the participants’ focus on the available evidence.

The somewhat inconsistent findings regarding the emergence of adversarial allegiance in the present study align with prior research by Sauerland et al. [17] and Arbiyah et al. [16]. While Sauerland et al. [17] were able to induce adversarial allegiance, Arbiyah et al. [16] were not, despite using the same materials and retention letters. This discrepancy underscores the challenges inherent in reliably inducing cognitive biases within a laboratory setting. One possible explanation for the inconsistent findings in the present study is that the Evidence Score may capture a more implicit measure of adversarial allegiance, whereas the credibility ratings reflect participants’ explicit evaluations. Given that credibility assessments in Germany usually do not require expert witnesses to quantify the credibility of a statement on a numerical scale from 1 to 100, participants may have consistently defaulted to the legal principle of *in dubio pro reo* when assigning their ratings across all groups. This interpretation would explain the lack of group differences and is supported by the observation that credibility ratings were consistently low across all groups ranging from *M* = 19.00 to *M* = 31.55.

Interestingly, exploratory analyses revealed that participants retained by the defense perceived the case evaluation as significantly easier than those retained by the court, who found the evaluation most difficult. Meanwhile, participants retained by the accessory prosecution fell between these two groups in terms of perceived difficulty and did not differ significantly from either. Participants retained by the court received a retention letter and phone call protocol that emphasized both evidence supporting and evidence challenging the credibility of the witness statement, thus conveying a more nuanced and balanced view of the case. In contrast, participants retained by the defense were exposed only to information that was critical of the statement’s credibility, creating a simplified and one-sided portrayal of the case. This framing may have led these participants to adopt the defense’s perspective, consistent with the observed adversarial allegiance, and to perceive the evaluation as considerably easier compared to participants retained by the court, who were explicitly made aware of the case’s ambiguity. The absence of significant group differences for participants retained by the accessory prosecution may be attributed to their limited familiarity with this retaining party, which could have prompted a more cautious or questioning stance. Additionally, expert witnesses are generally trained to apply falsification similar to the *in dubio pro reo* principle. This tendency in combination with a more cautious stance due to the unfamiliarity with the rentaining party may have somewhat reduced the impact of the one-sided information presented by the accessory prosecution, at least to the extent that participants did not perceive the case evaluation as significantly easier than those retained by the court.

On the other hand, the applied evaluation method did not produce a statistically significant effect on credibility ratings nor the Evidence Score. Specifically, participants utilizing the ACH method did not differ in their assessments from those employing the traditional approach commonly used in Germany, within the control group. This finding is contrary to initial expectations but aligns with previous studies that reported no significant debiasing effects of the ACH method [27,29,31]. Regarding the Evidence Score, and on a strictly descriptive level, it even appears that participants retained by the defense may have exhibited an increased focus on evidence supporting their retaining party’s position when applying the ACH method. This pattern may suggest that the ACH method has the potential to exacerbate certain cognitive biases, in this case adversarial allegiance. However, this finding did not reach statistical significance and should therefore be interpreted with caution. Nonetheless, it is consistent with earlier studies that reported at least partially detrimental effects of the ACH method on recognizing the diagnosticity and intercorrelation of the available evidence [30,33]. Further research is needed to examine the impact of the ACH method on evaluations and decision-making when used as a debiasing tool across different contexts.

The results also indicate that the application of the standard procedure used in Germany – which involves the evaluation and falsification-based elimination of alternative hypotheses in a relatively unstructured manner – was similarly insufficient to mitigate the effects of adversarial allegiance, as reflected in the Evidence Score, at least within the context of the present study. However, it is worth noting that while the sample in the present study possessed sufficient prior knowledge in credibility assessments, only some participants had previous practical experience serving as expert witnesses for credibility assessments in court. Although the observed adversarial allegiance did not yet result in statistically significant group differences in the credibility ratings in the present study, the expected biased pattern emerged at a descriptive level within the control group – hence, the group following the standard procedure. It is possible that differences in the evaluation of statement credibility would become more pronounced in real-life settings involving stronger biasing conditions, such as deeper involvement in the case, time pressure, and financial dependency – particularly within adversarial legal systems. While the biasing retaining parties examined in the present study do not typically occur within Germany’s inquisitorial legal system, where expert witnesses are usually appointed by the court, the present findings align with those reported by Sauerland et al. [17] as well as Arbiyah et al. [16], both of whom also failed to demonstrate the debiasing effects of strategies that have shown efficacy in other contexts. These strategies include, for example, the systematic evaluation of alternative hypotheses and two-sided instructions [e.g., 1,12]. Further research is needed to understand why existing debiasing strategies have thus far proven ineffective in this particular context and to identify alternative approaches that may more effectively mitigate adversarial allegiance.

The present study has several limitations. First, conducting the study online may have limited participants’ engagement with the case material, potentially contributing to the reduced presence of bias with respect to the credibility ratings. However, the use of manipulation- and attention-check questions, which screened out participants who failed to carefully review the retention letter and the case file, helped maintain a high standard of data quality.

Second, participants in this study had neither prior experience with nor received formal training for the application of the ACH method. However, they were systematically guided through steps 2–5 of the ACH method, ensuring the consistent application of each of these steps. Importantly, all participants possessed prior knowledge in credibility assessments within the German legal system. As such, they were familiar with the use of alternative hypotheses and a falsification-based approach, an approach that can be counterintuitive and challenging for laypersons. Given that the evaluation and falsification-based elimination of alternative hypotheses are central components of the ACH method, the participants in this study can be regarded as sufficiently experienced to apply the method effectively when provided with structured guidance. Additionally, a pilot study by Mierzejewski [35], which followed a similar procedure and instructions, found no additional advantage from incorporating a practice case and feedback. Nonetheless, it cannot be ruled out that the ACH method may be more effective in minimizing the observed adversarial allegiance in the Evidence Scores when applied by individuals who have received more extensive training in its use. Accordingly, future research should examine the potential moderating effects of training on the effectiveness of the ACH method.

Third, in relation to the aforementioned limitation, it could be argued that not all participants were currently practicing as independent expert witnesses in credibility assessment and that the sample therefore does not fully represent this population. However, the majority of the sample were fully qualified psychologists, most of whom held a master’s degree in psychology (59.7%) and several participants held PhDs or formal certifications as legal psychologists. Only a small minority of the sample consisted of psychology students whose highest reported qualification was a bachelor’s degree or a high school diploma (18.9% in total). It is important to note that, in Germany, there are no formal statutory requirements for serving as an expert witness in credibility assessment beyond holding a master’s degree (or equivalent) in psychology. At the same time, there is broad professional consensus that an additional certification or training in legal psychology is essential for competent practice in this field. A formal certification in legal psychology is offered by the Federation of German Psychologists’ Associations. However, due to the comparatively small size of this highly specialized field this certification is currently held by only a limited number of practitioners (March 2026: *N* = 150). Psychologists seeking to obtain this certification typically complete structured postgraduate training such as additional master’s programs or accredited professional seminars in legal psychology. During this advanced training phase, psychologists may already assist in the preparation of expert reports and are generally familiar with the standard procedures of credibility assessments. The majority of participants in the present study were at this stage of their professional development. To ensure sufficient domain-specific knowledge, eligibility was further verified through background knowledge questions assessing participants’ familiarity with established credibility assessment methodology. The high number of participants excluded based on eligibility criteria (*n* = 88), including individuals with master’s degrees in legal psychology, underscores the strictness of this study’s screening measures which was defined by the authors based on their theoretical, practical, and teaching experience in the field of credibility assessment. While restricting participation exclusively to formally certified legal psychologists would have been desirable, the limited number of such practitioners in Germany makes this infeasible for adequately powered experimental research. Accordingly, an approximation was necessary. Taken together, these considerations suggest that the final sample possessed sufficient familiarity with the procedures involved in credibility assessments within the German legal context to support the ecological validity of the study.

Fourth, although it is not unheard of in Germany, it remains uncommon for the accessory prosecution to retain its own expert witness to conduct credibility assessments. In this study, the accessory prosecution was selected as the retaining party because the state prosecution is typically regarded as a more neutral actor in legal proceedings, which might have failed to elicit adversarial allegiance. Due to participants’ limited familiarity with being retained by the accessory prosecution, it may have been more challenging for them to immerse themselves fully in the case and its context. Nevertheless, evidence of adversarial allegiance was observed in the comparison between the accessory prosecution and the defense with respect to the Evidence Score. Furthermore, participants retained by the accessory prosecution did not perceive the case evaluation as more difficult than participants in the other conditions.

Lastly, building on the previous point, it could be argued that it is important to distinguish between participants whose case acceptance may be shaped by financial or strategic considerations (e.g., consistently working for either the defense or the prosecution) and those who align with a party because they genuinely endorse a particular interpretation of the evidence, as such pre-existing tendencies can confound the study results. While this distinction is highly relevant in adversarial contexts, it is considerably less applicable to credibility assessments conducted within the German inquisitorial legal system. In Germany, expert witnesses are typically commissioned either (a) during the investigative phase by the prosecution when the decision to bring charges remains open, or (b) during the trial phase by the court. In the latter case, the defense may request an assessment, but the court generally appoints the expert witness, and direct retention by the defense is uncommon. Moreover, expert witnesses typically decide whether to accept a case before receiving the case materials, limiting the possibility of case selection based on pre-existing beliefs about guilt or innocence. Herein also lies a strength of this study as the context of the German inquisitorial legal system allows for adversarial allegiance to be examined without confounding effects from pre-existing alignment with or preference for either adversarial retaining party in this study. Moreover, participants were randomly assigned to retaining-party conditions, thereby preventing any systematic self-selection effects. Consistent with this, dropout rates following disclosure of the retaining party were low and did not differ meaningfully across conditions (defense: *n* = 1; accessory prosecution: *n* = 0; court: *n* = 3).

To conclude, the absence of a significant debiasing effects in the present study raises questions regarding the utility of the ACH method as a debiasing strategy in the context of credibility assessments. Due to the lack of explicit guidelines for evaluating and weighing the available evidence, the ACH method may function primarily as a structured framework that facilitates, rather than mitigates, potentially biased case assessments, an issue previously highlighted by Chang et al. [24]. At the same time, an outright rejection of ACH as an effective debiasing method would be premature at this stage. Nevertheless, the continued lack of supportive empirical findings across different contexts underscores the need for further research. Future studies should examine the conditions under which the ACH method may serve as an effective debiasing strategy as well as the factors that may contribute to its limited impact in certain settings. Such evaluations would benefit from using larger samples, involving participants with prior training and experience in applying the ACH method, and employing more immersive in-person settings, which may enhance engagement with the case material and the method itself.

## References

[1] Vredeveldt A, van Rosmalen EAJ, van Koppen PJ, Dror IE, Otgaar H. Legal psychologists as experts: Guidelines for minimizing bias. Psychology, Crime & Law. 2022;30(7):705–29. doi: 10.1080/1068316x.2022.2114476

[2] Dror IE, McCormack BM, Epstein J. Cognitive bias and its impact on expert witnesses and the court. Judges Journal. 2015;54(4):8–15.

[3] Dror IE. Cognitive and human factors in expert decision making: Six fallacies and the eight sources of bias. Anal Chem. 2020 June 16;92(12):7998–004. doi: 10.1021/acs.analchem.0c00704 32508089

[4] Murrie DC, Boccaccini MT, Guarnera LA, Rufino KA. Are forensic experts biased by the side that retained them?. Psychol Sci. 2013;24(10):1889–97. doi: 10.1177/0956797613481812 23969777

[5] Murrie DC, Boccaccini MT. Adversarial allegiance among expert witnesses. Annu Rev Law Soc Sci. 2015;11(1):37–55. doi: 10.1146/annurev-lawsocsci-120814-121714

[6] Findley KA, Scott MS. The multiple dimensions of tunnel vision in criminal cases. Wisconsin Law Review. 2006;2006(2):291–397.

[7] Chevalier CS, Boccaccini MT, Murrie DC, Varela JG. Static-99R reporting practices in sexually violent predator cases: Does norm selection reflect adversarial allegiance?. Law Hum Behav. 2015;39(3):209–18. doi: 10.1037/lhb0000114 25495715

[8] Edens JF, Cox J, Smith ST, DeMatteo D, Sörman K. How reliable are Psychopathy Checklist-Revised scores in Canadian criminal trials? A case law review. Psychol Assess. 2015;27(2):447–56. doi: 10.1037/pas0000048 25486503

[9] Lloyd CD, Clark HJ, Forth AE. Psychopathy, expert testimony, and indeterminate sentences: Exploring the relationship between Psychopathy Checklist‐Revised testimony and trial outcome in Canada. Leg Criminol Psychol. 2010;15(2):323–39. doi: 10.1348/135532509X468432

[10] Neal TMS, Lienert P, Denne E, Singh JP. A general model of cognitive bias in human judgment and systematic review specific to forensic mental health. Law Hum Behav. 2022;46(2):99–120. doi: 10.1037/lhb0000482 35191729

[11] McAuliff BD, Arter JL. Adversarial allegiance: The devil is in the evidence details, not just on the witness stand. Law Hum Behav. 2016;40(5):524–35. doi: 10.1037/lhb0000198 27243362 PMC5036989

[12] Oberlader VA, Quinten L, Schmidt AF, Banse R. How can I reduce bias in my work? Discussing debiasing strategies for forensic psychological assessments. Prof Psychol Res Pract. 2025. doi: 10.1037/pro0000615

[13] Lord CG, Lepper MR, Preston E. Considering the opposite: A corrective strategy for social judgment. J Pers Soc Psychol. 1984;47(6):1231–43. doi: 10.1037//0022-3514.47.6.1231 6527215

[14] Otgaar H, de Ruiter C, Howe ML, Hoetmer L, van Reekum P. A Case Concerning Children’s False Memories of Abuse: Recommendations regarding expert witness work. Psychiatr Psychol Law. 2016;24(3):365–78. doi: 10.1080/13218719.2016.1230924 31983961 PMC6818307

[15] van Koppen PJ, Mackor AR. A scenario approach to the simonshaven case. Top Cogn Sci. 2020;12(4):1132–51. doi: 10.1111/tops.12429 31155856 PMC7687160

[16] Arbiyah N, Otgaar H, Sauerland M, Rassin E, Maegherman E, Merckelbach H. The use of alternative scenarios in assessing the reliability of victims’ statements. Psychology, Crime & Law. 2023;31(3):291–308. doi: 10.1080/1068316x.2023.2236274

[17] Sauerland M, Otgaar H, Maegherman E, Sagana A. Allegiance bias in statement reliability evaluations is not eliminated by falsification instructions. Zeitschrift für Psychologie. 2020;228(3):210–5. doi: 10.1027/2151-2604/a000416

[18] BGH. Wissenschaftliche Anforderungen an aussagepsychologische Begutachtungen (Glaubhaftigkeitsgutachten). Prax Rechtspsychologie. 1999;9(2):46–125.

[19] Volbert R, Steller M. Is this testimony truthful, fabricated, or based on false memory? Credibility assessment 25 years after. Eur Psychol. 2014;19(3):207–20. doi: 10.1027/1016-9040/a000200

[20] Heuer RJ. Psychology of Intelligence Analysis. Washington, DC: U.S. Central Intelligence Agency, Center for the Study of Intelligence. 1999.

[21] Heuer RJ. The future of “alternative analysis”. http://www.pherson.org/wp-content/uploads/2013/06/04.-Future-of-Alternative-Analysis_FINAL.pdf. 2007.

[22] Heuer RJ. How does analysis of competing hypotheses (ACH) improve intelligence analysis. http://www.pherson.org/wp-content/uploads/2013/06/06.-How-Does-ACH-Improve-Analysis_FINAL.pdf. 2005.

[23] Jones N. Critical epistemology for analysis of competing hypotheses. Intelligence and National Security. 2018;33(2):273–89. doi: 10.1080/02684527.2017.1395948

[24] Chang W, Berdini E, Mandel DR, Tetlock PE. Restructuring structured analytic techniques in intelligence. Intelligence and National Security. 2018;33(3):337–56. doi: 10.1080/02684527.2017.1400230

[25] Dhami MK, Belton IK, Mandel DR. The analysis of competing hypotheses in intelligence analysis. Appl Cogn Psychol. 2019;33(6). doi: 10.1002/acp.3550

[26] Wilcox J, Mandel DR. Critical review of the analysis of competing hypotheses technique: Lessons for the intelligence community. Intelligence and National Security. 2024;:1–22. doi: 10.31234/osf.io/an32t

[27] Lehner PE, Adelman L, Cheikes BA, Brown MJ. Confirmation bias in complex analyses. IEEE Trans Syst, Man, Cybern A. 2008;38(3):584–92. doi: 10.1109/tsmca.2008.918634

[28] Mandel DR, Karvetski CW, Dhami MK. Boosting intelligence analysts’ judgment accuracy: What works, what fails?. Judgm decis mak. 2018;13(6):607–21. doi: 10.1017/s1930297500006628

[29] Whitesmith M. Cognitive bias in intelligence analysis: Testing the analysis of competing hypotheses method. Edinburgh: Edinburgh University Press. 2020.

[30] Karvetski CW, Mandel DR, Irwin D. Improving probability judgment in intelligence analysis: From structured analysis to statistical aggregation. Risk Anal. 2020;40(5):1040–57. doi: 10.1111/risa.13443 32065440

[31] Maegherman E, Ask K, Horselenberg R, Koppen PJ. Test of the Analysis of Competing Hypotheses in legal decision-making. Appl Cogn Psychol. 2021;35(1):62–70. doi: 10.1002/acp.3738

[32] Otzipka J, Volbert R. The analysis of competing hypotheses in legal proceedings. Appl Cogn Psychol. 2025;39(5):e70115. doi: 10.1002/acp.70115

[33] Karvetski CW, Mandel DR. Coherence of probability judgments from uncertain evidence: Does ACH help?. Judgm decis mak. 2020;15(6):939–58. doi: 10.1017/s1930297500008159

[34] Faul F, Erdfelder E, Buchner A, Lang A-G. Statistical power analyses using G*Power 3.1: Tests for correlation and regression analyses. Behav Res Methods. 2009;41(4):1149–60. doi: 10.3758/BRM.41.4.1149 19897823

[35] Mierzejewski MJ. Testing the ‘Analysis of Competing Hypotheses’ in the Context of Criminal Investigations. Freie Universität Berlin. 2022.

